# Measuring What Matters in Trial Operations: Development and Validation of the Clinical Trial Site Performance Measure

**DOI:** 10.3390/jcm14196839

**Published:** 2025-09-26

**Authors:** Mattia Bozzetti, Alessio Lo Cascio, Daniele Napolitano, Nicoletta Orgiana, Vincenzina Mora, Stefania Fiorini, Giorgia Petrucci, Francesca Resente, Irene Baroni, Rosario Caruso, Monica Guberti

**Affiliations:** 1 Department of Biomedicine and Prevention, University of Rome Tor Vergata, 00133 Rome, Italy; mattia.bozzetti@asst-cremona.it; 2 Direction of Health Professions, La Maddalena Cancer Center, 90146 Palermo, Italy; locascio.alessio@lamaddalenanet.it; 3 CEMAD, Fondazione Policlinico Gemelli IRCCS, 00168 Rome, Italy; vincenzina.mora@policlinicogemelli.it; 4 Department of Cardiovascular, Neural and Metabolic Sciences, Istituto Auxologico Italiano, IRCSS San Luca Hospital, 20149 Milan, Italy; s.fiorini@auxologico.it; 5 Operative Research Unit of Orthopaedic and Trauma Surgery, Fondazione Policlinico Universitario Campus Bio-Medico, 00128 Rome, Italy; g.petrucci@policlinicocampus.it; 6 Department of Oncohematology, Presidio Infantile Regina Margherita, Azienda Ospedaliero Universitaria Città della Salute e della Scienza di Torino, 10126 Turin, Italy; francesca.resente@unito.it; 7 Health Professions Research and Development Unit, IRCSS Policlinico San Donato, 20097 San Donato Milanese, Italy; irene.baroni@grupposandonato.it (I.B.); rosario.caruso@unimi.it (R.C.); 8 Department of Biomedical Sciences for Health, University of Milan, 20141 Milan, Italy; 9 Allied Health Professions Directorate, Istituto Ortopedico Rizzoli, 40136 Bologna, Italy; monica.guberti@ior.it

**Keywords:** clinical trials, site performance, real-world data, advanced statistical methods, regulatory challenges, operational metrics, quality monitoring, clinical epidemiology, clinical trial site performance measure

## Abstract

**Background/Objectives:** The execution of clinical trials is increasingly constrained by operational complexity, regulatory requirements, and variability in site performance. These challenges have direct implications for the reliability of trial outcomes. However, standardized methods to evaluate site-level performance remain underdeveloped. This study introduces the Clinical Trial Site Performance Measure (CT-SPM), a novel framework designed to systematically capture site-level operational quality and to provide a scalable short form for routine monitoring. **Methods:** We conducted a multicenter study across six Italian academic hospitals (January–June 2025). Candidate performance indicators were identified through a systematic review and expert consultation, followed by validation and reduction using advanced statistical approaches, including factor modeling, ROC curve analysis, and nonparametric scaling methods. The CT-SPM was assessed for structural validity, discriminative capacity, and feasibility for use in real-world settings. **Results:** From 126 potential indicators, 18 were retained and organized into four domains: Participant Retention and Consent, Data Completeness and Timeliness, Adverse Event Reporting, and Protocol Compliance. A bifactor model revealed two higher-order dimensions (participant-facing and data-facing performance), highlighting the multidimensional nature of site operations. A short form comprising four items demonstrated good scalability and sufficient accuracy to identify underperforming sites. **Conclusions:** The CT-SPM represents an innovative, evidence-based instrument for monitoring trial execution at the site level. By linking methodological rigor with real-world applicability, it offers a practical solution for benchmarking, resource allocation, and regulatory compliance. This approach contributes to advancing clinical research by providing a standardized, data-driven method to evaluate and improve performance across networks.

## 1. Introduction

Clinical trials are central to therapeutic innovation and evidence-based medicine [1] but remain among the most complex and resource-intensive forms of research [2,3]. They face increasing complexity, rising costs, and growing expectations driven by regulatory demands, safety requirements, and pressures from multiple stakeholders [4,5]. From an epidemiological perspective, these challenges directly affect the validity, transparency, and reproducibility of trial findings, raising the need for methodological innovation in how trials are monitored and assessed.

Regulatory bodies have sought to address performance variability through Good Clinical Practice (GCB) guidelines and the promotion of Risk-Based Monitoring (RBM), which encourages adaptive oversight based on objective metrics [6]. However RBM implementation remains limited, largely due to the absence of validated and reproducible performance indicators [7,8]. Given their information and regulation-intensive nature, clinical trials could benefit substantially from digital tools such as electronic data capture (EDC) systems and RBM supported by advanced analytics, which have the potential to improve operational efficiency, data quality, regulatory compliance, and organizational preparedness [9]. Yet, uneven adoption of these technologies across sites and regions perpetuates performance disparities and risks exacerbating digital inequalities, with both managerial and ethical implications [10,11].

Within clinical epidemiology, this evolution highlights the principles of *Quality by Design* (QbD) that are strengthened by the ICH E6 (R3) GCP guidelines [12], which emphasize prospectively building quality into clinical trials rather than relying solely on retrospective monitoring. When combined with QbD, digital tools can serve as enablers of bias control, ensuring that trial conduct maintains internal validity and supports the reproducibility of results across multicentre settings.

Site performance is a decisive factor for trial success [1,13,14]. Problems such as poor recruitment, delayed visits, low data quality, or protocol deviations compromise both epidemiological validity and ethical accountability, often leading to trial delays or premature termination [15,16,17]. Recruitment challenges are a major contributor, with approximately 11% of cardiovascular trials on ClinicalTrials.gov ending prematurely due to poor accrual, resulting in significant ethical and economic consequences [18]. The absence of standardized, validated performance metrics exacerbates these issues, and around 30% of trials fail or remain unpublished [19], often due to preventable operational problems rather than scientific flaws [8] leading to wasted resources and eroding patient trust [20,21].

Current monitoring practices frequently rely on subjective or arbitrary indicators [22], making it difficult to identify underperforming sites, implement targeted improvement strategies, or compare performance across studies [23,24]. Additional barriers include the sustainability of digital solutions over time, institutional and governance constraints, legal and ethical risks, cybersecurity concerns, and an incomplete understanding of the contextual factors that enable or hinder digital transformation in healthcare [25,26]. Implementing a structured risk management approach could enhance quality and safety [27].

Furthermore, without a shared definition of “high performance” and standardized thresholds, assessments remain inconsistent, affecting site selection and monitoring [28]. Leveraging real-world data and advanced statistical approaches could support predictive models for more objective, scalable, and reproducible evaluations [29].

Within this context, the creation of a validated, scalable tool for the objective measurement of clinical trial site performance represents not only a managerial improvement but also a methodological advance in clinical epidemiology. Such a tool can reduce preventable failures, improve the efficiency of resource deployment, and uphold the ethical responsibility to ensure that research investments translate into meaningful and reproducible health outcomes.

## 2. Materials and Methods

### 2.1. Aims

This study aimed to develop and validate the Clinical Trial Site Performance Measure (CT-SPM) as a methodological innovation in clinical epidemiology. Specifically, the objectives were to:

(i) Construct a psychometrically robust framework capturing core domains of site-level operational performance relevant to trial validity and reproducibility;
(ii) Identify a parsimonious core indicator set through Mokken Scale Analysis to enable scalable and transparent monitoring;
(iii) Establish evidence-based cut-offs to distinguish optimal from suboptimal site performance, thereby supporting bias control and risk-based monitoring; and;
(iv) Design the instrument for seamless integration into a digital application, facilitating real-time benchmarking across multicentre trials and alignment with principles of QbD and modern clinical research governance.

### 2.2. Setting

A cross-sectional study (January–June 2025) was conducted in six Italian research centers (IRCCSs, university and public hospitals). A pre-specified protocol was published [30].

### 2.3. Inclusion Criteria

All the clinical trials at participating centers were eligible, aiming to maximize generalizability and minimize phase/status bias.

### 2.4. Instrument Development

**Figure 1:**
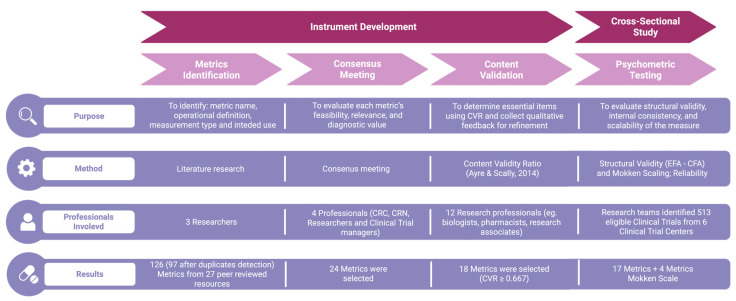
The figure illustrates the four sequential phases of the Clinical Trial Site Performance Measure (CT-SPM) development process: Metrics Identification, Consensus Meeting, Content Validation, and Psychometric Testing. For each phase, the diagram reports: the main purpose (e.g., identifying relevant metrics, evaluating content validity, assessing psychometric properties); the method used (e.g., literature review, expert panel discussions, Content Validity Ratio calculation, exploratory and confirmatory factor analysis); the types and number of professionals involved, and; the results obtained (e.g., number of metrics retained or refined). Abbreviations: CT-SPM = Clinical Trial Site Performance Measure; CRC = Clinical Research Coordinator; CRN = Clinical Research Nurse; CVR = Content Validity Ratio; EFA = Exploratory Factor Analysis; CFA = Confirmatory Factor Analysis [31].

The development of the CT-SPM followed a structured, multi-phase process designed to ensure both scientific rigor and practical relevance (Figure 1). Developed instruments are available in Table S1 and Metric selection in Table S3. The CT-SPM was designed as a two-tier instrument, comprising a short-form core set for universal application and a scalable full form that can be tailored to the monitoring needs of specific trials.

The retained metrics were structured as behaviorally anchored items suitable for self-assessment using a five-point Likert scale (1 = “Not frequent” to 5 = “Highly frequent”). To ensure accurate and comprehensive evaluation, the CT-SPM was completed jointly by all team members involved in each clinical trial. This consensus-based approach minimized individual bias and captured shared operational experience. Each item response reflected the frequency with which specific practices or events occurred during trial conduct.

### 2.5. Variables Collected

A wide range of study-level and site-level variables were collected by the research team to avoid recall bias to characterize the context and to support exploratory analyses of associations with CT-SPM performance scores. Descriptive variables included the department or therapeutic area (e.g., oncology, haematology, cardiology), the type of treatment administered (single vs. multiple contact), trial phase (I–IV), translational nature (clinical vs. translational), and study design (e.g., randomized controlled trial, basket trial, platform trial, cluster trial). Information was also gathered on whether special procedures were applied during the study (e.g., GCP training, informed consent and documentation processes, adverse event reporting, complex data management procedures), and whether any centralized processes were implemented (e.g., centralized randomization, data management, or monitoring). In addition, a binary outcome variable was collected to serve as a reference criterion for performance classification. This variable was derived from a study-level question in which research teams were asked to indicate whether the trial, in their judgment, had deviated in a significant and unsatisfactory way from expected performance standards (1 = “Yes” and 0 = “No”).

### 2.6. Statistical Analyses

Data analysis proceeded in multiple stages. Descriptive statistics were first computed for each item, with results reported as means (M) and standard deviations (SD). Patterns of missing data were examined with Little’s missing completely at random (MCAR) test confirmed that data were MCAR [32]. To minimize potential loss of statistical power due to missingness, predictive mean matching (PMM) was used for imputation, implemented via the mice package [33]. Given the expected presence of ceiling and floor effects observed in the distribution of several items, a logarithmic transformation was applied to reduce skewness and improve the approximation to normality. All analyses were conducted using R (version 4.3.3) [34] with statistical significance set at *p* < 0.05.

#### 2.6.1. Psychometric Testing

Psychometric evaluation proceeded in multiple stages. Prior to exploratory factor analysis (EFA) analysis, data suitability was assessed using the Kaiser–Meyer–Olkin (KMO) measure of sampling adequacy, with a minimum acceptable value set at 0.60, and Bartlett’s test of sphericity, with statistical significance established at *p* < 0.05. To cross-validate the factor structure, a split-sample approach was employed; the total sample was randomly divided into two subgroups, with approximately 60% allocated to EFA. Confirmatory Factor Analysis (CFA) was subsequently conducted on the second subsample to test the structural validity of a bifactor model composed of one general factor and two orthogonal specific factors. Model fit was evaluated using multiple indices: the chi-square test, Comparative Fit Index (CFI ≥ 0.95), Tucker–Lewis Index (TLI ≥ 0.95), Root Mean Square Error of Approximation (RMSEA ≤ 0.06), and Standardized Root Mean Square Residual (SRMR ≤ 0.08) [35]. The Bayesian Information Criterion (BIC) was also used for model comparison and to evaluate relative model parsimony.

Reliability was evaluated using several indices. McDonald’s omega (ω) and hierarchical omega (ω_h_) were calculated to estimate internal consistency, with ω ≥ 0.70 indicating acceptable reliability. To assess unidimensionality, explained common variance (ECV) and ω_h_ values were examined, with thresholds of ≥0.70 used to support the use of a total score [36]. A lower ECV or ω_h_ suggested the need for scoring based on separate subscales.

#### 2.6.2. Mokken Scaling

Mokken Scale Analysis (MSA) was conducted to develop a nonparametric short-form version of the CT-SPM. MSA comprises two main components: (a) an automated item selection procedure that organizes ordinal items into Mokken scales and (b) analytical techniques for testing the assumptions of nonparametric item response theory (IRT) models. Mokken models are based on three key assumptions: unidimensionality, local independence, and latent monotonicity (Ark, 2012 [37]; Mokken, 2011 [38]). Unidimensionality indicates that all items within a scale measure a single underlying construct (θ). Local independence implies that responses to one item are not influenced by responses to others. Latent monotonicity assumes that the probability of endorsing higher response categories increases monotonically with the latent trait.

The Monotone Homogeneity Model (MHM) incorporates all three assumptions and allows respondents to be meaningfully ranked along the latent dimension based on their total scores [39]. The Double Monotonicity Model (DMM) adds the requirement of invariant item ordering (IIO), whereby the rank order of item difficulty remains constant across all levels of the latent trait. Establishing such item hierarchies is essential to ensure generalizability and interpretability across populations.

To evaluate scalability, Loevinger’s scalability coefficients were computed. These include item-pair (Hij), item (Hi), and scale-level (Hs) coefficients. Coefficients range from 0 to 1, with higher values indicating greater discriminative power. A scale is considered weak if Hs is between 0.30 and <0.40, moderate if between 0.40 and <0.50, and strong if ≥0.50. Items with Hi values < 0.30 or those that violate IIO assumptions were considered for removal. The internal consistency of the scale was assessed using the Molenaar–Sijtsma reliability coefficient (ρ) [39]. Assumptions of monotonicity were tested using coefficient H and associated violation metrics. Monotonicity ensures that item scores increase proportionally with the underlying trait level, indicating consistent ordering along the latent continuum.

#### 2.6.3. Calculation of Standardized Scores

For each metric and each factor, the standardized score was calculated using the formula:

$$\left(\frac{\left(\sum x-n\right)}{\left(n\times (5-1)\right)}\right)\times 100$$

where x represents the score attributed to each individual item, while ∑x denotes the total sum of item scores within a given factor. The variable *n* indicates the number of items that compose the factor. The constant 4 reflects the range of the Likert scale. The resulting value is then multiplied by 100 to express the standardized score on a 0–100 scale, facilitating direct comparisons across factors.

#### 2.6.4. Performance Cut-Off Definition

Receiver Operating Characteristic (ROC) curve analysis was conducted to evaluate the discriminative capacity of the CTSPM and short form. The Area Under the Curve (AUC) was used as a summary index of diagnostic accuracy. Optimal cut-off values were determined using Youden’s J statistic, identifying the threshold that maximized the trade-off between sensitivity and specificity in classifying trial protocols as adequate or inadequate in terms of site performance.

#### 2.6.5. Sample Size Calculation

Sample size requirements for the MSA were determined using a power-based simulation approach to ensure sufficient statistical power to detect meaningful scalability under the assumptions of the Mokken model [40]. The simulation parameters included a baseline of 24 items, an initial sample size of *n* = 200 participants, and *n* = 100 Monte Carlo simulations per iteration. A target power of 0.80 was set, with the minimum acceptable scale scalability coefficient (Hs) fixed at 0.40. A maximum of 20 iterations was specified.

At each iteration, the average achieved power was calculated. If the desired power threshold was not met, the sample size was increased by 10 participants and simulations were repeated. This process continued until the power estimate reached at least 0.80 or the maximum number of iterations was exhausted. Results from these simulations indicated that a minimum sample size of 400 was required to achieve adequate power within 20 iterations, while increasing to 600 participants was necessary for simulations conducted over 40 iterations. These findings guided the target sample size for the MSA and ensured adequate model stability and generalizability of the short-form scale.

### 2.7. Deployment

A proof-of-concept digital dashboard was developed using Streamlit (Python 3.13) to demonstrate real-time computation of CT-SPM composite scores and workload indices. The prototype ingested structured trial, patient, and staffing data from local .csv sources (simulating EDC/CTMS API outputs), performed on-the-fly psychometric scoring, and rendered interactive dashboards for site-level performance monitoring, protocol complexity assessment, and resource allocation.

## 3. Results

The study included a total of 513 clinical trials. The majority of studies were conducted in oncology settings (46.0%), followed by gastroenterology (17.3%) and cardiology-pneumology (5.8%). Other specialties represented included allergology, neurology, rheumatology, and others, each accounting for less than 5% of the total. Regarding the type of treatment, 93.2% of the studies involved active interventions, while 6.4% were observational studies, either single or multiple contact surveys.

In terms of study phase, a large portion were Phase III (38.0%), followed by Phase II (21.5%) and Phase I (10.2%). Most studies did not involve translational research (52.3%), although 47.4% did. The most common study design was randomized controlled trials (RCTs), representing 43.1% of the total, followed by prospective cohort studies (29.6%) and observational non-interventional studies (5.7%). Vast majority of studies enrolled between 1 and 50 subjects (91.8%). As for study duration, most had a length of 1–3 years (86.9%), followed by 6 months–1 year (8.5%) and 0–6 months (3.0%). Finally, 90.4% of studies reported no protocol deviations, while 9.4% acknowledged the presence of deviations.

### 3.1. Phase 2: Psychometric Testing

Factorability of the data was supported by the KMO (overall MSA = 0.78; range 0.63–0.91 and a significant Bartlett’s test of sphericity (χ^2^(153) = 5970.97, *p* < 0.001). Parallel analysis suggested retaining five factors. Subsequently, an EFA was performed using ML estimation with oblimin rotation to allow for correlated factors. The five-factor solution accounted for 60.3% of the total variance (Table S2).

However, during the CFA, the fifth factor and Item 11 showed unstable fit and did not meaningfully contribute to the model. Their inclusion negatively affected model parsimony and interpretability. Therefore, the fifth factor and Item 11 were excluded from the final CFA model, resulting in a more robust and theoretically coherent four-factor solution.

#### 3.1.1. Structural Validity

The model demonstrated satisfactory fit to the data (χ^2^_(64)_ = 1131.29, *p* < 0.001; CFI = 0.983; TLI = 0.974; RMSEA = 0.079, 90% CI [0.071, 0.090]; SRMR = 0.066) accounting for 64.6% of the total variance. The measurement model comprises four first-order latent factors and two second-order latent factors. Factor 1 (F1), labelled “Participant Retention and Consent,” includes variables related to participant dropout, consent, and withdrawal rates. Factor 2 (F2), labelled “Data Completeness and Timeliness,” encompasses indicators of data quality, timeliness of Case Report Form entries, and rates of missing data. Factor 3 (F3), labelled “Adverse Events Reporting,” represents the frequency and accuracy of adverse event documentation. Factor 4 (F4), labelled “Protocol Compliance,” captures variables related to protocol deviations and violations.

**Figure 2:**
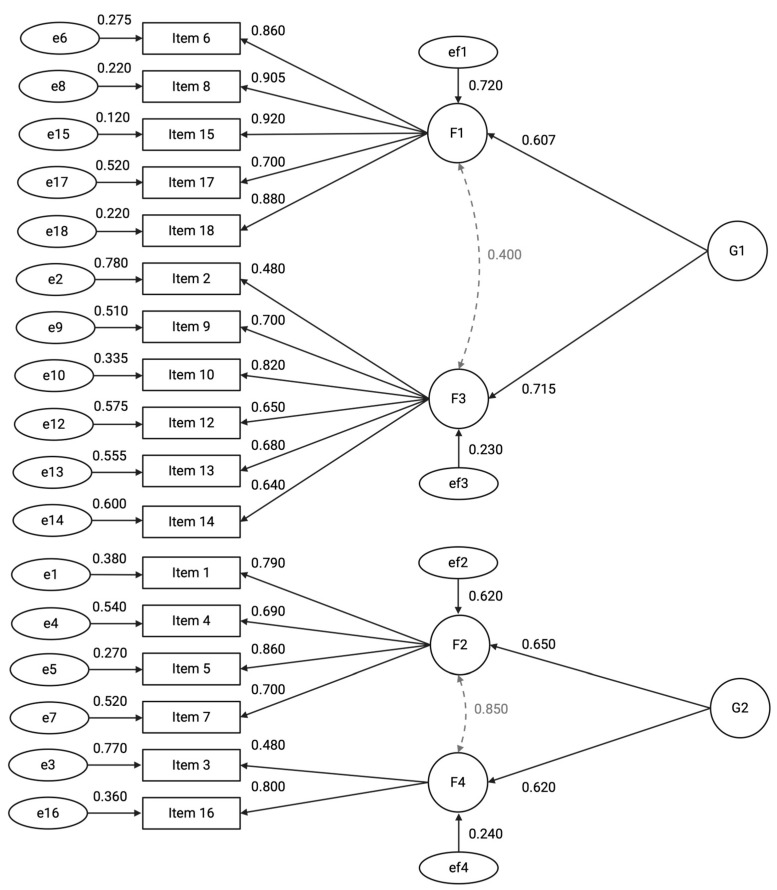
Path diagram of the hierarchical factor model illustrating relationships between latent constructs and observed variables. First-order factors (F1–F4) are indicated by circles, each measured by multiple observed items (rectangles) with standardized factor loadings shown on single-headed arrows. Second-order factors (G1 and G2) influence the first-order factors, with standardized regression coefficients labeled on the paths. Residual variances for observed items and first-order factors are represented by error terms (ellipses) with corresponding estimates. Bidirectional arrows denote covariances among first-order factors. All parameter estimates are fully standardized. For clarity, residual covariances between items—specified in the model—are not depicted in the diagram.

At the higher level, the two second-order factors represent broader constructs. Factor G1, labelled “Participant Retention and Adverse Event Monitoring,” influences F1 and F3, reflecting participant-related outcomes and safety monitoring. Factor G2, labelled “Data Quality and Protocol Adherence,” influences F2 and F4, representing overarching data integrity and adherence to trial protocols (Figure 2). To improve model fit several residual covariances were specified based on high modification indices and theoretical plausibility. Specifically, residual correlations were added between Items 17–16 (r = 0.42)*,* Items 17–4 (r = 0.39)*,* Items 1–3 (r = 0.34)*,* Items 6–4 (r = 0.41), and Items 4–13 (r = 0.44).

#### 3.1.2. Reliability

Reliability analyses and the factor structure are summarized in Table 1.

### 3.2. Phase 3: Mokken Scaling

#### 3.2.1. Automatic Item Selection Procedure

The analysis resulted in the selection of a four-item scale composed of Item9, Item10, Item12, and Item17. The overall scalability coefficient of the scale was H = 0.489, SE = 0.027. Hi were as follows: Item9, Hi = 0.563; Item10, Hi = 0.495; Item12, Hi = 0.557; and Item17, Hi = 0.504. These values exceeded the conventional threshold of 0.30, supporting their inclusion. Item16, which showed Hi = 0.216, was excluded due to insufficient scalability.

#### 3.2.2. Monotonicity

The item Hi were consistently above the acceptable threshold, ranging from Hi = 0.548 for Item17 to Hi = 0.631 for Item9. Minor violations of monotonicity were detected for Item12 (1 violation) and Item17 (2 violations). No violations were reported for Item9 or Item10. Overall, the pattern of results suggests that the monotonicity assumption was largely met across the selected items.

Monotonicity was evaluated for the refined four-item Mokken scale composed of Item9, Item10, Item12, and Item17. Hi were consistently above the conventional threshold of 0.30, ranging from Hi = 0.548 for Item17 to Hi = 0.631 for Item9, indicating adequate scalability. Minor violations of monotonicity were detected for Item12 (1 violation) and Item17 (2 violations), while no violations were observed for Item9 or Item10. These findings suggest that the assumption of monotonicity was largely upheld across the selected items.

The results of the IIO analysis are presented in Table 2. The total HT coefficient was 0.461, reflecting a moderate to strong level of invariant ordering. Item-level HT values were uniformly 0.46, except for Item9, which exhibited a substantially stronger ordering structure (HT = 0.82). During the backward selection procedure, Item17 was flagged for removal due to two violations of invariant ordering. Nonetheless, given its acceptable scalability (Hi = 0.53) and theoretical relevance, it was retained in the final model. No other items demonstrated critical violations. Based on scalability, monotonicity, and invariant ordering, the final scale retained four items—Item9, Item10, Item12, and Item17.

Internal consistency of the refined four-item scale was supported by ρ = 0.818, Cronbach’s α = 0.778, and Guttman’s λ_2_ = 0.781. All coefficients supported the psychometric adequacy of the refined scale (Table 3).

### 3.3. Phase 3: Defining Performance Cut-Off

The cut-off values varied across factors, reflecting differences in scoring distributions. The discriminative ability of the factors, measured by the AUC, ranged from poor to moderate, with the short form showing the highest AUC = 0.628. These results are summarized in Table 3.

**Table 3:** Cut-offs for each sub-scale.

Factors	M (SD)	Cutoff (Youden’s)	AUC
Participant Retention and Consent (F1)	61.30 (25.70)	22.5	0.583
Data Completeness and Timeliness (F2)	71.80 (21.20)	46.9	0.528
Adverse Events Reporting (F3)	62.50 (10.90)	64.6	0.557
Protocol Compliance (F4)	82.50 (17.30)	6.25	0.293
Participant Retention and Adverse Event Monitoring (G1)	61.90 (14.90)	55.2	0.581
Data Quality and Protocol Adherence (G2)	77.10 (16.70)	60.9	0.562
Short Form (Mokken Scale)	40.27 (24.33)	59.38	0.628

Note. M = Mean; SD = Standard Deviation; AUC = Area Under the Curve.

## 4. Discussion

This study offers a methodological contribution to clinical epidemiology by introducing and validating an RBM instrument that addresses the persistent absence of validated, reproducible, and scalable indicators for site-level performance.

The CT-SPM was conceived as a two-tier instrument: (i) a scalable full form that can be tailored to study-specific monitoring needs and (ii) short-form core set for universal use across interventional and observational studies. Beyond operational management, the framework is conceived to strengthen internal validity, transparency, and reproducibility of clinical trials across multicentre settings, core priorities for contemporary clinical epidemiology [12]. By leveraging an integrated psychometric approach, this tool goes beyond isolated site metrics [5,22,41,42,43] to deliver a multifactorial framework aligned with current priorities in clinical epidemiology—such as bias control and robust reporting standards.

The CT-SPM revealed a coherent four-factor structure encompassing critical domains of trial execution. Factor 1 (Retention and Consent) reflects participant engagement and protocol adherence, anchored by indicators like consent rate. Factor 2 (Data Timeliness and Completeness) captures CRF delays and missing data, key metrics of data quality and workflow efficiency. Factor 3 (AEs) reflects both the accuracy and frequency of safety reporting, with item convergence on SAEs and data concordance supporting its validity. AE underreporting is often linked to procedural issues rather than clinical factors [44]. Factor 4 (Protocol Compliance) includes items on violations and deviations, revealing systemic issues in eligibility and coordination. While overlapping with participant behavior, these reflect operational weaknesses. Consistent with prior frameworks [45], protocol adherence is vital for trial integrity, with deviations known to impact outcomes significantly [46,47].

The emergence of two higher-order dimensions as Retention & Safety and Data Integrity & Protocol Adherence, provides a parsimonious lens for targeted quality interventions and pre-specified decision rules at the site level [48].

The MSA distils performance into four robust indicators: query frequency, SAE accuracy, outcome-data queries, protocol violations—balancing feasibility with measurement rigor. While the AUC indicates moderate discriminative accuracy, the short form enables transparent, continuous monitoring and rapid screening consistent with risk-proportionate oversight and pre-trial site selection [7,49,50]. Item-level behavior aligns with known quality levers: high query frequency is a sensitive proxy for workflow inefficiencies [51]; SAE concordance captures safety-reporting fidelity where under-reporting often reflects procedural rather than clinical causes [44,52]; primary-outcome queries track endpoint integrity [53] and protocol violations signal compliance risks that can degrade trial integrity [45,46,47].

Despite minor monotonicity (2) and IIO issues, it showed acceptable scalability (Hi = 0.504) and theoretical relevance, given the known link between violations and trial integrity [45]. Together, these four items define a coherent “operational reliability” construct, supported by moderate-to-strong scalability and few monotonicity breaches. Their grouping under the Data/Protocol Integrity factor aligns with modern trial quality frameworks [48], enabling comprehensive yet focused site assessment. This concise scale balances process and outcome metrics, favoring objectivity and feasibility. It supports risk-based monitoring and pre-trial site selection by flagging data quality and protocol compliance issues, providing actionable insights for sponsors [7,49,50,54].

The CT-SPM is designed for digital integration. Alignment with EDC/CTMS and hospital data flows enables near real-time analytics, supports pre-specified RBM triggers, and facilitates transparent audit trails. This positions the tool within a broader Quality-by-Design and good clinical practice ecosystem, moving beyond retrospective monitoring towards proactive bias control through design-embedded quality safeguards [12]. Looking ahead, integration with real-world data sources and advanced computational methods can extend CT-SPM from descriptive monitoring to predictive and prescriptive decision support, for example, using supervised models to anticipate site under-performance and central statistical monitoring to detect anomalous patterns [29,55]. Such extensions should be accompanied by governance and fairness safeguards to mitigate automation bias and digital inequities [10,11,25].

Our performance framework has reporting and reproducibility implications. A minimal reporting set for site-performance methods and results including item definitions, scoring rules, thresholds, and decision logic—would enhance methodological transparency and comparability across trials and settings. Public availability of the instrument, scoring code, and a data dictionary would further support reusability and replication, addressing calls to reduce avoidable waste and improve the value of clinical research [20,52].

### 4.1. Strengths and Limitations

The CT-SPM provides a transparent, psychometrically validated and reproducible instrument that spans participant- and data-facing domains central to trial validity. The combination of a multidimensional full scale and a parsimonious short form enables scalable deployment for risk-based monitoring and Quality-by-Design applications across heterogeneous settings.

Some limitations must be acknowledged. First, analyses are cross-sectional, limiting causal interpretation of performance–outcome relationships. Second, cut-offs were calibrated against a subjective site-level criterion, introducing potential reference bias; future work should adopt objective outcomes and pre-registered decision rules. Third, the moderate AUC of the short form and the relatively lower discrimination of the protocol-compliance domain suggest that context-sensitive thresholds, control-chart approaches, or time-updated indicators may better capture dynamic risk. Fourth, the study was conducted in Italian sites; cross-national validation is needed to support generalizability.

### 4.2. Implications for Future Research

Although the CT-SPM provides a validated structure and a concise short form, accounting for 64.6% of the total variance, it remains clear that despite explaining a substantial proportion of the variance, the instrument does not yet capture the full complexity of what constitutes good performance across all trial contexts. Future investigations should therefore aim to validate the instrument internationally, prospectively testing it across different healthcare systems and therapeutic areas. The indicator set should be iteratively expanded and refined, with the development of context-specific metrics and the calibration of dynamic or control-chart–based thresholds that better reflect changes in site performance over time. Further research should also evaluate predictive and prescriptive approaches, such as supervised models and central statistical monitoring, to detect under-performance and anomalous patterns. In addition, the potential use of the CT-SPM as a design-embedded covariate (for example through stratification, adjustment, or hierarchical modelling with site-level random effects) deserves careful assessment. Finally, integration with RWD should be accompanied by explicit governance and fairness safeguards to mitigate automation bias and digital inequities. These steps will help move the CT-SPM from a proof-of-concept toward a next-generation performance score able to encompass the broader and more nuanced dimensions of trial quality.

## 5. Conclusions

The CT-SPM provides a structured, validated tool for assessing clinical trial site performance. Its dual format supports flexible application in diverse monitoring contexts. Further validation will strengthen its utility for evidence-based oversight and site management.

## Figures and Tables

**Table 1:** Reliability indices for the latent factors.

Factors	Omega Total (ω)	Omega Hierarchical (ω_h_)	Explained Common Variance (ECV)
Participant Retention and Adverse Event Monitoring (G1)	0.73	0.59	0.42
Data Quality and Protocol Adherence (G2)	0.68	0.53	0.40
Participant Retention and Consent (F1)	0.82	0.70	
Data Completeness and Timeliness (F2)	0.75	0.64	
Adverse Events Reporting (F3)	0.70	0.60	
Protocol Compliance (F4)	0.65	0.58	

**Table 2:** Summary of invariant item ordering (IIO) analysis for the final four-item Mokken scale.

Items	H	#ac	#vi	#vi/#ac	maxvi	sum	sum/#ac	tmax	#tsig	crit	Selection	HT	Rho
Item9	0.63	4	1	0.25	0.18	0.18	0.05	1.14	0	1.96	0	0.55	0.82
Item10	0.53	5	0	0.00	0.00	0.00	0.00	0.00	0	1.96	0	0.60	
Item12	0.58	5	1	0.20	0.44	0.62	0.12	2.75	1	1.96	1	0.54	
Item17	0.53	4	2	0.50	0.57	0.70	0.18	5.05	1	1.96	2	0.63	

Note. H = item scalability coefficient, indicating the strength of the item’s contribution to the hierarchical scale; #ac = number of active score groups; #vi = number of violations of invariant item ordering (IIO); #vi/#ac = proportion of violations relative to the number of active score groups; maxvi = maximum single violation; sum = total violations observed across score groups; sum/#ac = average number of violations per score group; tmax = maximum t-value observed for any violation; #tsig = number of statistically significant violations (*p* < 0.05); crit = critical t-value threshold at α = 0.05; Selection = backward selection flag (1 = selected after backward elimination); HT = Loevinger’s HT coefficient, representing the degree of invariant item ordering; Rho = Molenaar–Sijtsma reliability coefficient.

## Data Availability

The datasets used and/or analysed during the current study are available from the corresponding author on reasonable request.

## Supplementary Materials

The following supporting information can be downloaded at: https://www.mdpi.com/article/10.3390/jcm14196839/s1, Table S1: Clinical Trial Performance Metrics; Table S2: Exploratory Factor Analysis; Table S3: Clinical Trial Performance Metrics -Short Form (Mokken Scale).

## Appendix A

Performance Working Group: Valentina Accinno, Caterina Fanali, Ester Udugbor, Marica Di Curcio, Eleonora Ribaudi, Stefania Colan-tuono, Francesca Garibaldi, Corinne Tavano, Valeria Altamura, Alessia Leonetti, Maria Anna Teberino, Cristina Graziani, Luciana Giannone, Roberta Savini, Giulia Wlderk, Daniele Privitera, Silvia Elettra Re-vere, Giulia Paglione, Giada De Angeli, Nathasha Samali Udugampolage, Miriam Angolani.
