# Supplementary material for: Measuring What Matters in Trial Operations: Development and Validation of the Clinical Trial Site Performance Measure
Source: J Clin Med. 2025 Sep 26;14(19):6839. doi: 10.3390/jcm14196839 (PMC12524836; doi:10.3390/jcm14196839)
Supplement: Supplementary file 1 [file jcm-14-06839-s001.zip › jcm-3881565-supplementary.pdf]

Table S1. Clinical Trial Performance Metrics

	Factor	Item	Metrics
Participant Retention and Adverse Event Monitoring (G1)	Participant Retention and Consent (F1)	Item 6	Number of enrolled participants who discontinued the study before trial closure divided by the total number of enrolled participants
		Item 8	Number of eligible individuals who provided consent to participate divided by the total number of eligible individuals
		Item 15	Number of randomized participants who withdrew consent at the site divided by the total number of randomized participants
		Item 17	Number of major protocol deviations divided by the total number of enrolled participants
		Item 18	Number of participants with missing primary outcome data divided by the total number of participants expected to provide such data
	Adverse Events Reporting (F3)	Item 2	Number of reported serious adverse events matching entries in the trial database or follow-up documentation divided by the total number of serious adverse events reported
		Item 9	Number of queries issued by the central trial team regarding primary outcome data divided by the total number of randomized participants at the site
		Item 10	Number of randomized participants reporting at least one adverse event divided by the total number of randomized participants
		Item 12	Number of endpoint data entries consistent with protocol requirements in the EDC system divided by the total number of endpoint data entries
		Item 13	Number of collected biological samples compliant with protocol specifications divided by the total number of collected samples
		Item 14	Number of protocol violations related to eligibility criteria and randomization divided by the total number of protocol violations

*Continues

	Factor	Item	Metrics
Data Quality and Protocol Adherence (G2)	Data Completeness and Timeliness (F2)	Item 1	Number of serious adverse events reported among participants with completed follow-up divided by the total number of participants with completed follow-up
		Item 4	Number of CRFs entered after the predefined timeline divided by the total number of CRFs expected
		Item 5	Number of trials where database lock occurred within the planned timeline after the last participant visit divided by the total number of completed trials
		Item 7	Number of CRFs with manual queries due to missing, out-of-range, or inconsistent data divided by the total number of CRFs reviewed
	Protocol Compliance (F4)	Item 3	Number of randomized participants with one or more protocol violations divided by the total number of randomized participants
		Item 16	Number of randomized participants who initiated the assigned intervention divided by the total number of randomized participants

Table S2. Exploratory Factor Analysis

Item	Factor 1	Factor 2	Factor 3	Factor 4	Factor 5
Item1	-0.186	**0.688**	-0.093	0.272	-0.134
Item2	0.126	-0.066	**0.363**	**0.506**	0.237
Item3	0.017	0.110	0.079	**0.756**	-0.133
Item4	0.040	**0.866**	0.128	-0.120	0.124
Item5	-0.063	**0.704**	0.070	0.259	0.104
Item6	**0.593**	0.232	0.256	0.045	-0.230
Item7	0.145	**0.673**	-0.150	0.054	-0.227
Item8	**0.738**	0.014	0.270	0.040	-0.194
Item9	0.224	-0.221	**0.641**	0.287	0.008
Item10	0.066	0.036	**0.724**	-0.049	0.102
Item11	-0.142	0.051	0.081	-0.064	**0.707**
Item12	-0.094	0.187	**0.533**	0.044	-0.032
Item13	0.127	0.273	**0.556**	-0.230	0.115
Item14	-0.233	0.022	**0.595**	-0.191	-0.317
Item15	**1.000**	0.122	-0.073	-0.075	0.046
Item16	-0.134	0.261	-0.071	**0.585**	0.066
Item17	**0.714**	-0.234	0.000	-0.100	-0.182
Item18	**0.961**	-0.116	-0.035	0.095	0.068
**Statistic**	**Factor 1**	**Factor 2**	**Factor 3**	**Factor 4**	**Factor 5**
SS loadings	3.599	2.555	2.229	1.536	0.930
Proportion Var	0.200	0.142	0.124	0.085	0.052
Cumulative Var	0.200	0.342	0.466	0.551	0.603

Table S3: Clinical Trial Performance Metrics -Short Form (Mokken Scale)

Factor	Item	Metrics
Mokken Scale	Item9	Number of queries issued by the central trial team regarding primary outcome data divided by the total number of randomized participants at the site
	Item10	Number of randomized participants reporting at least one adverse event divided by the total number of randomized participants
	Item12	Number of endpoint data entries consistent with protocol requirements in the EDC system divided by the total number of endpoint data entries
	Item17	Number of major protocol deviations divided by the total number of enrolled participants
